# A nomogram for predicting the risk of postoperative fever in elderly patients undergoing endoscopic submucosal dissection of the upper gastrointestinal tract

**DOI:** 10.1097/MD.0000000000036438

**Published:** 2023-12-15

**Authors:** Zhixiang Xu, Jing Zhuang, Xin Zhu, Jun Yao

**Affiliations:** a The Affiliated People’s Hospital of Jiangsu University, Zhenjiang, China; b Department of Gastroenterology, The Affiliated People’s Hospital of Jiangsu University, Zhenjiang, China.

**Keywords:** elderly, endoscopic submucosal dissection, fever, nomogram, risk factors

## Abstract

To explore the risk factors of postoperative fever in elderly patients undergoing endoscopic submucosal dissection (ESD). A total of 439 patients who met the inclusion criteria were enrolled in this study and randomly divided into training (n = 311) and validation (n = 128) cohorts at a ratio of 7:3. Independent risk factors were screened by single-factor and multiple-factor logistic regression analyses, and a nomogram was established using them. The nomogram was evaluated using receiver operating characteristic curve analysis, decision curve analysis, and calibration plot using the “rms” package in R software (R4.2.1). The study included 439 patients. Female (*OR* = 2.55, 95% *CI*: 1.5–4.33), diabetes (*OR* = 2.38, 95% *CI*: 1.17–4.85), operation time (*OR* = 1.01, 95% *CI*: 1–1.02) were lesion located in the esophagus (*OR* = 2.37, 95% *CI*: 1.44–3.88), maximum tumor diameter (*OR* = 1.3, 95% *CI*: 1.07–1.57), and placement of a urinary catheter (*OR* = 7.09, 95%*CI*: 1.43–35.17) were independent risk factors for postoperative fever in elderly ESD patients (*P* < .05). Female sex, diabetes, lesions located in the esophagus, lesion size, operation time, and placement of a urinary catheter are risk factors for postoperative fever in ESD patients, and patients with these risk factors should be vigilant for postoperative fever and receive appropriate treatment.

## 1. Introduction

Endoscopic submucosal dissection (ESD) is an endoscopic procedure for submucosal resection and has become one of the primary treatments for early gastric cancer and gastric esophageal mucosal lesions.^[1,2]^ This procedure has the advantages of being minimally invasive, with fast recovery, minimal trauma, and functional preservation, making it more advantageous than traditional surgery. However, ESD is a difficult procedure that requires a high level of technical and professional knowledge, and may lead to complications such as bleeding, perforation, and fever, poses challenges, particularly in elderly patients.^[3,4]^ Recognizing and mitigating the risk factors for postoperative fever is crucial, as fever can be indicative of serious complications such as infection, can prolong hospital stay, and may lead to further invasive procedures and antibiotic use, thus negating the benefits of a minimally invasive approach. Furthermore, in the geriatric population, the prompt identification and management of fever are essential due to their reduced physiological reserves and the increased risk of morbidity and mortality. Consequently, our study aims to enhance perioperative care by identifying predictors of fever specific to the elderly, facilitating preoperative risk stratification, and enabling individualized management plans. Through these efforts, we strive to maximize the clinical outcomes and safety profile of ESD in this vulnerable cohort.

## 2. Materials and methods

### 2.1. Patients and data collection

Retrospective data collection was conducted on 439 elderly patients who underwent ESD at the First People Hospital of Zhenjiang between January 2017 and January 2022. The inclusion criteria required patients to undergo upper gastrointestinal ESD during the specified time period and to have complete clinical data. The exclusion criteria were age < 65 years, immunodeficiency, preoperative fever, concomitant tumors in other parts of the body, and incomplete clinical data.

Observational indicators included patient characteristics such as sex, age, BMI, history of diabetes, smoking history, alcohol history, lesion characteristics including lesion location, lesion size, depth of infiltration, and surgery time, and postoperative management, including placement of gastric and urinary catheters.

### 2.2. Operative procedures

Under endoscopic guidance, a specific tool was used to demarcate a 0.5 to 1.0 cm range around the lesion to determine the dissection area. Subsequently, submucosal fluid is injected around the lesion to create a bulge, facilitating surgical removal of the lesion through dissection and excision under the submucosa until complete removal is achieved. During dissection, close attention is paid to bleeding at the surgical site, and measures, such as hemostatic drugs, electrocoagulation, and hemostatic forceps, are promptly taken if necessary. All the procedures were performed by experienced gastroenterologists.

### 2.3. Statistical analysis

Statistical analysis was conducted using the SPSS software (version 26.0). The normality of the data was verified using the Shapiro–Wilk test. Normally distributed quantitative data are presented as mean ± standard deviation and analyzed using independent sample t-tests for intergroup differences. Non-normally distributed quantitative data are presented as *M (IQR*) and analyzed using the Mann–Whitney *U* test for intergroup differences. Categorical data are presented as the number of cases (percentage) and analyzed using the chi-square test for intergroup differences. Univariate and multivariate logistic regression analyses were used to identify independent risk factors for post-ESD fever, and these factors were used to establish a column-chart prediction model. The “rms” package in R (R4.2.1) was used to generate the column chart, and the model was evaluated using receiver operating characteristic curves, decision curve analysis (DCA), and calibration plots. Statistical significance was defined as *P* < .05.

## 3. Results

### 3.1. Comparison of clinical data between training cohort and validation cohort

This study aimed to investigate 439 patients aged 65 years or older who underwent ESD. The patients were randomly allocated into a modeling group consisting of 311 cases and a validation group consisting of 128 cases in a ratio of 7:3. The baseline characteristics of the 2 groups are presented in Table 1. No statistically significant differences were found between the 2 groups in terms of age, sex, BMI, smoking, drinking, diabetes, maximum tumor diameter, tumor location, operation time, submucosal infiltration, gastric tube, and urinary catheter use (*P* > .05). These findings indicated that the data characteristics of the training and validation sets were consistent and comparable.

**Table 1:** Comparison of clinical data between training cohort and validation cohort.

	Level	Overall	Training cohort	Validation cohort	*P*
n		439	311	128	
Age (M(IQR))		70.91 (4.51)	70 (7)	70 (6)	.475
BMI (M(IQR))		22.93 (2.93)	23 (4.1)	22.73 (4.2)	.109
Sex (%)					.344
	Male	284 (64.69)	206 (66.24)	78 (60.94)	
	Female	155 (35.31)	105 (33.76)	50 (39.06)	
Diabetes (%)					.931
	No	378 (86.10)	267 (85.85)	111 (86.72)	
	Yes	61 (13.90)	44 (14.15)	17 (13.28)	
Smoke (%)					.143
	No	320 (72.89)	220 (70.74)	100 (78.12)	
	Yes	119 (27.11)	91 (29.26)	28 (21.88)	
Drink (%)					.166
	No	358 (81.55)	248 (79.74)	110 (85.94)	
	Yes	81 (18.45)	63 (20.26)	18 (14.06)	
Tumor_size (M(IQR))		3.32 (1.37)	3 (4.1)	3.25 (2)	.268
Tumor_location (%)					.16
	Stomach	244 (55.58)	180 (57.88)	64 (50.00)	
	Esophagus	195 (44.42)	131 (42.12)	64 (50.00)	
Submucosal_infiltration (%)					.46
	No	407 (92.71)	286 (91.96)	121 (94.53)	
	Yes	32 (7.29)	25 (8.04)	7 (5.47)	
Operate_time (M(IQR))		87.31 (38.90)	80 (40)	80 (45)	.727
Stomach_tube (%)					.578
	No	219 (49.89)	152 (48.87)	67 (52.34)	
	Yes	220 (50.11)	159 (51.13)	61 (47.66)	
Urethral_catheter (%)					.804
	No	422 (96.13)	298 (95.82)	124 (96.88)	
	Yes	17 (3.87)	13 (4.18)	4 (3.12)	
Fever* (%)					.284
	No	194 (44.19)	143 (45.98)	51 (39.84)	
	Yes	245 (55.81)	168 (54.02)	77 (60.16)	

* T ≥ 37.5°C.

### 3.2. Univariate and multivariate analysis

Table 2 presents the results of the univariate and multivariate logistic regression models for the clinical characteristics of the modeling group. Univariate analysis showed that sex, operation time, maximum tumor diameter, tumor location, and indwelling urinary catheter were associated with postoperative fever after ESD. Factors with a *P* value <.1 were included in the multivariate analysis. The results of the multivariate analysis showed that female sex (*OR* = 2.55, 95%*CI*: 1.5–4.33), diabetes (*OR* = 2.38, 95%*CI*: 1.17–4.85), operation time (*OR* = 1.01, 95%*CI*: 1–1.02), tumor location in the esophagus (*OR* = 2.37, 95%*CI*: 1.44–3.88), maximum tumor diameter (*OR* = 1.3, 95%*CI*: 1.07–1.57), and indwelling urinary catheter (*OR* = 7.09, 95%*CI*: 1.43–35.17) were independent risk factors for postoperative fever in elderly patients undergoing ESD (*P* < .05).

**Table 2:** Univariate and multivariate analysis.

Characteristics	B	OR	Univariate analysis			Multivariate analysis		
			95%CI	*P*	B	OR	95%CI	*P*
Age	0.02	1.02	0.97–1.07	.44				
BMI	-0.038	0.96	0.89–1.04	.347				
Diabetes	0.577	1.78	0.91–3.47	.091	0.868	2.38	1.17–4.85	.017
Drink	-0.242	0.78	0.45–1.37	.391				
Operate_time	0.007	1.01	1–1.01	.022	0.008	1.01	1–1.02	.031
Sex	0.606	1.83	1.13–2.97	.014	0.935	2.55	1.5–4.33	.001
Smoke	-0.259	0.77	0.47–1.26	.299				
Stomach_tube	-0.046	0.95	0.61–1.49	.839				
Submucosal_infiltration	-0.8	0.45	0.19–1.05	.065				
Tumor_location	0.714	2.04	1.29–3.24	.002	0.861	2.37	1.44–3.88	.001
Tumor_size	0.253	1.29	1.08–1.53	.005	0.26	1.3	1.07–1.57	.008
Urethral_catheter	1.597	4.94	1.08–22.65	.04	1.959	7.09	1.43–35.17	.016

The factors with *P* <.1 in the univariate analysis were used for multifactor analysis (*Stepwise regression*).

### 3.3. Model construction and verification

**Figure 1.**
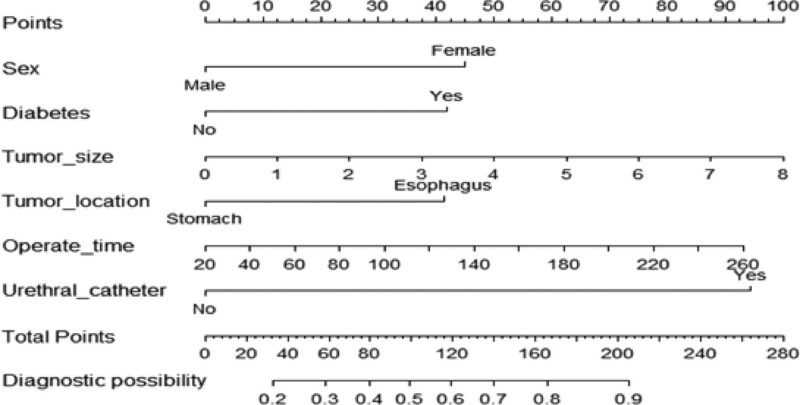
A nomogram predicting the risk of fever after ESD surgery. A straight line was drawn on the horizontal axis to the score value to obtain the score of each independent risk factor. The score of each independent risk factor was added together to obtain the total score. According to the total score, the risk axis was located to obtain the probability of fever after ESD operation. ESD = endoscopic submucosal dissection.

This study established a nomogram model (Fig. 1) for predicting postoperative fever in elderly patients undergoing ESD, based on 6 independent risk factors screened by multivariate analysis: sex, diabetes, operation time, lesion location, maximum tumor diameter, and indwelling urinary catheter. Each factor was assigned a different score, which was then added to obtain the patient total score. The corresponding probability of postoperative fever was calculated from the total score.

**Figure 2.**
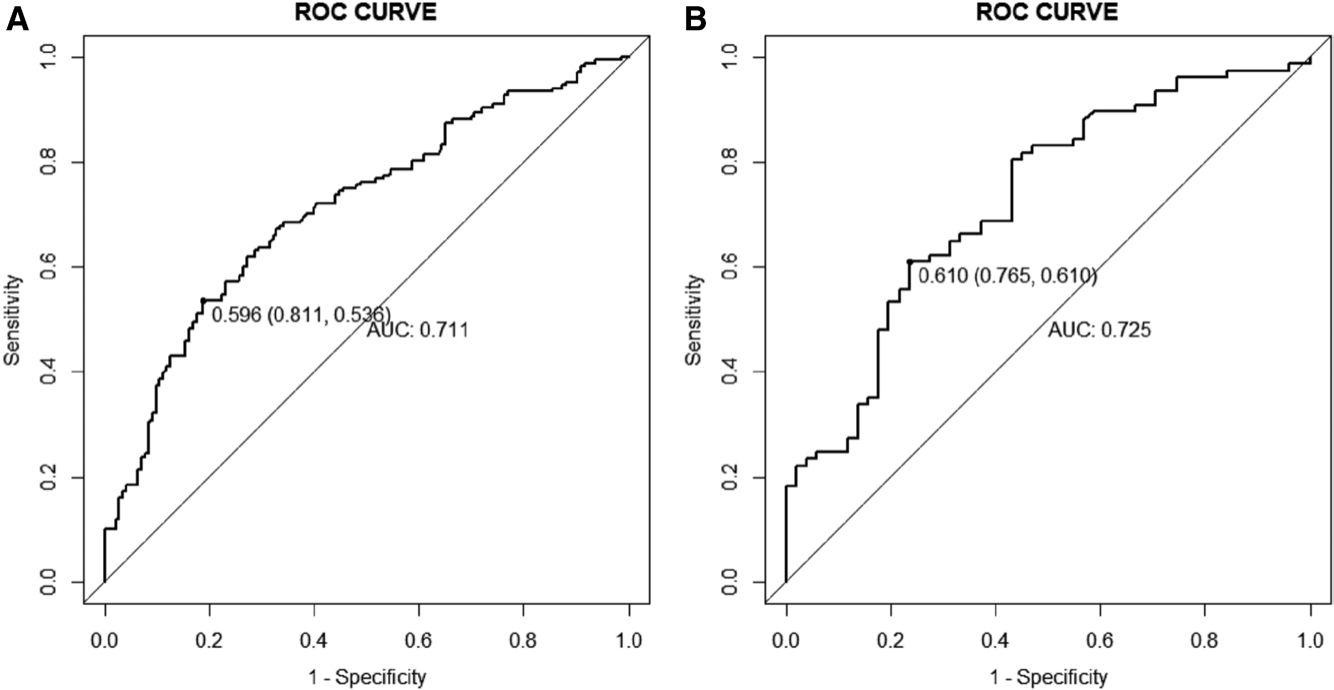
The ROC curve of training cohort (A) and validation cohort (B). A: AUC0.711 B: AUC0.725. ROC = receiver operating characteristic.

**Figure 3.**
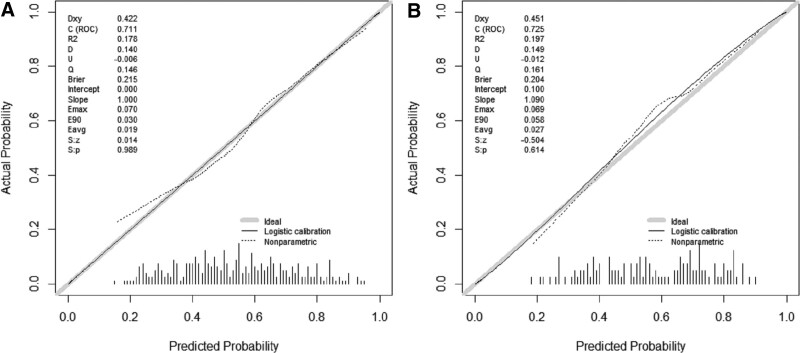
Calibration Plot. We assessed calibration by creating a calibration plot and calculating the calibration slope. Training cohort (A) and validation cohort (B) have better calibration.

**Figure 4.**
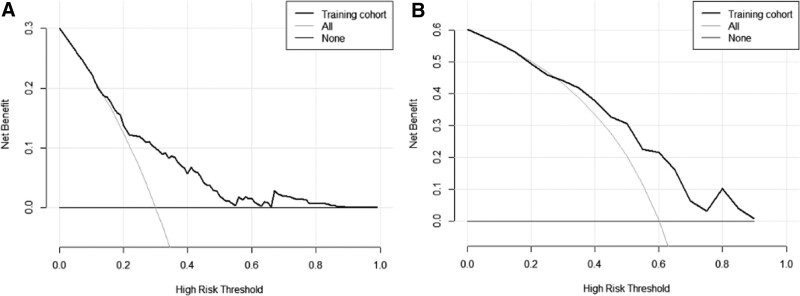
Decision curve analysis (DCA) of training cohort (A) and validation cohort (B). The y-axis represents the net benefit, which is the difference between the number of true positives and the number of false positives weighted by a factor that reflects the relative importance of correctly identifying true positives versus avoiding false positives. The x-axis represents the threshold probability, which is the minimum probability required for a patient to be considered at high risk of the outcome of interest.

Receiver operating characteristic curve analysis (Fig. 2A) indicated that the AUC of the nomogram model was 0.711, indicating good discrimination and prediction ability. The calibration curve showed that the predicted probability of postoperative fever was consistent with the actual probability of postoperative fever (Fig. 3A). According to the DCA, the nomogram model had clinical utility when the threshold was set between 18% and 50% (Fig. 4A).

Validation with the validation group data showed that the AUC of the nomogram model was 0.725, indicating a good discriminative ability (Fig. 2B). DCA and calibration curves of the nomogram model showed good clinical efficacy (Figs. 3B and 4B).

## 4. Discussion

Our study offers several novel insights into post-ESD fever management in the elderly. Unlike previous research, our nomogram provides a personalized risk assessment tool, enabling clinicians to predict postoperative fever with greater precision. This facilitates early interventions tailored to individual patient profiles, thereby potentially reducing complication rates and improving outcomes. Moreover, our investigation uniquely focuses on an elderly demographic, a group typically underrepresented in ESD research, hence addressing a significant knowledge gap. The identified independent risk factors, such as the association of urinary catheter placement with a sevenfold increase in fever risk, underscore the importance of cautious postoperative care in this population. This information is vital for refining perioperative strategies and underscores the necessity of vigilant monitoring in elderly ESD patients.

With the development of endoscopic technology, endoscopic resection has become the standard treatment for early gastric cancer and gastric mucosal lesions.^[5,6]^ Compared with endoscopic mucosal resection, ESD has a higher overall and curative resection rate for esophageal lesions, but the incidence of adverse events also increases,^[7]^ which may be related to the complexity of the ESD procedure. After ESD, we often focus on severe adverse events such as bleeding and perforation, while postoperative fever is also common. Although a series of studies have been conducted on the factors influencing post-ESD fever, controversy remains. The empirical use of antibiotics is usually applied to patients with fever in clinical practice to reduce the incidence of adverse events after ESD and improve clinical efficacy.^[8,9]^ However, some studies have suggested that the low incidence of post-ESD bacteremia makes the use of antibiotics unnecessary.^[10,11]^ A nomogram is a graphical tool used to visualize and calculate the relationships between multiple variables. It is a simple, fast, and intuitive method that can be used for analysis, prediction, and decision-making.^[12]^ This study aimed to provide guidance for clinical medication use by constructing a risk prediction model for post-ESD fever in elderly patients.

This study demonstrates that the location of the lesion in the esophagus, maximum tumor diameter, duration of surgery, female sex, placement of a urinary catheter, and diabetes mellitus are independent risk factors for postoperative fever after ESD. The reasons for this are as follows: Lesion location in the esophagus: due to the thinner wall of the esophagus, the difficulty of the operation is increased, and repeated flushing of the wound is required during the operation, combined with the patient general anesthesia, which increases the incidence of aspiration pneumonia. There are reports that postoperative pneumonia is associated with the continuous administration of propofol anesthesia during surgery, and these factors can contribute to postoperative fever. Previous studies have reported a 30.3% incidence of postoperative fever after esophageal ESD in 446 patients.^[13]^ Other studies have shown that the incidence of postoperative fever after gastric ESD is between 13.04% and 24.8%,^[14,15]^ consistent with the results of this study. Maximum tumor diameter: Larger tumor diameters often result in a larger range of ESD excision and wound surface, and the inflammatory response during healing may be one of the causes of fever. Clinically, for such patients, efforts should be made to minimize surgical wounds while ensuring complete tumor resection. Previous studies have also shown that lesion size is an independent risk factor for postoperative fever after colorectal ESD.^[16]^ Duration of surgery: The duration of surgery is a risk factor for surgery-related pneumonia,^[17]^ and prolonged ESD operations may delay wound healing and increase the risk of infection, leading to an increased incidence of postoperative fever. Female sex and urinary catheterization: In this study, female sex and placement of a urinary catheter were identified as risk factors for post-ESD fever. A study of 117 Japanese patients showed that female sex was an independent risk factor for post-gastric ESD fever.^[16]^ In this study, 2 patients developed postoperative urinary tract infections, both of whom were female and underwent urinary catheterization, suggesting that female physiological structures may be prone to urinary tract infections, and clinical physicians should be cautious when performing urinary catheterization. Diabetes mellitus: Clinically, diabetes mellitus is considered to be closely related to the occurrence and aggravation of infections. Previous studies have shown that hyperglycemia reduces neutrophil bactericidal capacity by inhibiting migration, phagocytosis, and superoxide production, leading to decreased immunity and increased infection rates.^[18,19]^

In response to the independent risk posed by urinary catheterization, we advocate for a judicious approach to its use during ESD. The potential for catheter associated UTIs necessitates a critical evaluation of its necessity, favoring less invasive urine collection methods when feasible. For those requiring catheterization, strict adherence to aseptic techniques and prompt removal post-ESD could mitigate this risk. Additionally, our findings suggest the implementation of surveillance protocols for early detection and treatment of UTIs in postoperative ESD patients. By optimizing catheter management, we aim to preserve the minimally invasive nature of ESD and maintain its favorable risk profile.

## 5. Conclusions

In conclusion, the location of the lesion in the esophagus, maximum diameter of the tumor, duration of surgery, female sex, placement of a urinary catheter, and diabetes are independent risk factors for postoperative fever after ESD. The nomogram developed in this study can help predict the risk of postoperative fever after ESD, and clinical interventions should focus on these factors to reduce the risk of postoperative infections. However, this study has some limitations as it is a single-center retrospective study, and further studies with larger sample sizes and prospective external validation are needed.

## Acknowledgments

Thank you to all who contributed to the process of writing this article.

## Author contributions

**Data curation:** Xin Zhu.

**Investigation:** Xin Zhu.

**Methodology:** Jing Zhuang.

**Resources:** Zhixiang Xu, Jing Zhuang.

**Supervision:** Jun Yao.

**Writing – original draft:** Zhixiang Xu.

**Writing – review & editing:** Jun Yao.
